# Effects of statin therapy on mean platelet volume in patients with risk of cardiovascular diseases: a systematic review and meta-analysis

**DOI:** 10.1042/BSR20190180

**Published:** 2019-07-26

**Authors:** Shuaifei Ji, Babo Zhang, Xianda Wang, Heng Shi, Lixin Yu, Xiaocheng Wang

**Affiliations:** 1 School of basic medicine, Air Force Military Medical University, Xi’an, Shanxxi, China; 2 Center of Clinical Aerospace Medicine, School of Aerospace Medicine, Key Laboratory of Aerospace Medicine of Ministry of Education, Air Force Medical University, Xi’an, China

**Keywords:** mean platelet volume, meta analysis, Statins

## Abstract

Many studies have demonstrated the effects of statin therapy on platelet, but it is controversial that whether statin could reduce mean platelet volume (MPV) in patients with the risk of cardiovascular diseases. To further improve the clinical significance of MPV in those patients and explore new function of statin, we conducted this research. Relevant studies were selected by searching electronic databases (PubMed, Embase and Cochrane Library) and reference lists of related articles by hand. Two reviewers independently assessed eligibility and quality of the studies. Eventually, we included ten studies, a total of 1189 patients with the risk of cardiovascular diseases. Consolidating relevant data and comparing the changes of MPV before and after statin treatment, we found that statin could decrease MPV [standard mean difference (SMD) = −0.47 (−0.71–0.23)], which was statistically significant (*P*=0.0001). Subgroup analysis suggested that when ≥55 years, this decrease did not occur [SMD = −0.06 (−0.18, 0.06)]. Drug type, sample size, ethnicity, mean age and quality of included article were sources of heterogeneity. Therefore, statin therapy could reduce MPV significantly and exhibited antiplatelet activity, which is of great importance in clarifying the clinical significance of MPV in cardiovascular events and the prevention of cardiovascular events.

## Introduction

Mean platelet volume (MPV), reflecting the size of the platelets, is a potentially useful marker of platelet activity. Increased MPV level has been identified as an independent risk factor for cardiovascular diseases and vascular risk factors such as coronary heart disease [1], diabetes [2], smoking [3], hypertension [4], dyslipidemia [5] obesity [6] and atrial fibrillation [7].

Statin inhibits 3-hydroxy-3-methylglutaryl co-enzyme A reductase in the mevalonate pathway, simultaneously promotes the low density lipoprotein metabolism and the high density lipoprotein synthesis, which play an essential role in reducing the risk of cardiovascular events. In recent years, many studies have reported possible effects of statin on MPV, but the conclusions have not been uniform and are highly controversial. Some studies have linked statin to lower MPV [8–15], others have found no link [16,17]. So do statin affect MPV? Could MPV be an independent risk factor for patients taking statin with cardiovascular risk factors? Based on this controversial topic and these questions, we conducted a systematic review and meta-analysis to study the relationship between statin therapy and MPV, with a view to providing a reference for clinical practice.

## Materials and methods

### Literature search

This systematic review and meta-analysis are reported in accordance with the Preferred Items for Systematic Reviews and Meta-analysis (PRISMA) Statement. Literature was retrieved by formal search of electronic databases (PubMed, Embase and Cochrane Library) without date limitation. To achieve the maximum sensitivity of the search strategy, we used appropriated free text and thesaurus terms including “Hydroxymethylglutaryl CoA Reductase Inhibitors”, “statin”, “mean platelet volume”. We also searched reference lists of related articles by hand to obtain more studies. All studies were limited to English language and the retrieval strategy of Pubmed as follow: (((((Mean Platelet Volumes[Title/Abstract] OR Platelet Volume, Mean[Title/Abstract] OR Platelet Volumes, Mean[Title/Abstract] OR Volume, Mean Platelet[Title/Abstract] OR Volumes, Mean Platele[Title/Abstract]))) OR “Mean Platelet Volume”[Mesh])) AND (((((Hydroxymethylglutaryl CoA Reductase Inhibitors[Title/Abstract] OR Inhibitors, Hydroxymethylglutaryl-CoA Reductase[Title/Abstract] OR Reductase Inhibitors, Hydroxymethylglutaryl-CoA[Title/Abstract] OR Inhibitors, HMG-CoA Reductase[Title/Abstract] OR Inhibitors, HMG CoA Reductase[Title/Abstract] OR Reductase Inhibitors, HMG-CoA[Title/Abstract] OR HMG-CoA Reductase Inhibitors[Title/Abstract] OR HMG CoA Reductase Inhibitors[Title/Abstract] OR Statins, HMG-CoA[Title/Abstract] OR HMG-CoA Statins[Title/Abstract] OR Statins, HMG CoA[Title/Abstract] OR Inhibitors, Hydroxymethylglutaryl-CoA[Title/Abstract] OR Hydroxymethylglutaryl-CoA Inhibitors[Title/Abstract] OR Inhibitors, Hydroxymethylglutaryl CoA[Title/Abstract] OR Statins[Title/Abstract] OR Inhibitors, Hydroxymethylglutaryl-Coenzyme A[Title/Abstract] OR Hydroxymethylglutaryl-Coenzyme A Inhibitors[Title/Abstract] OR Inhibitors, Hydroxymethylglutaryl Coenzyme A[Title/Abstract])) OR (*statin[Title/Abstract] OR atorvastatin[Title/Abstract] OR rosuvastatin[Title/Abstract] OR pravastatin[Title/Abstract] OR simvastatin[Title/Abstract] OR statin[Title/Abstract]))) OR “Hydroxymethylglutaryl-CoA Reductase Inhibitors”[Mesh]).

### Inclusion and exclusion criteria

Inclusion criteria: (1) Patients with the risk of cardiovascular diseases, such as diabetes mellitus, dyslipidemia and hypertension; (2) The data must contain standard mean difference (SMD). If only median and interquartile range (IQR) provided, standard difference (SD) will be calculated according to the Cochrane manual equation: SD = IQR/1.359 [18]; (3) The article must provide baseline data. (4) Abstracts providing necessary data will be included in order to avoid bias.

Exclusion criteria: (1) Reviews, case reports, letters and unpublished studies; (2) Animal-based experiments; (3) Duplication of a previous publication; (4) *In vitro* studies; (5) Unrelated studies; (6) Not cardiovascular diseases.

### Data abstraction and quality assessment

Two authors (S.F.J. and B.B.Z.) independently extracted the original data. Disagreement was resolved by discussion. If the two authors could not reach a consensus, the result was reviewed by the third author (X.D.W.). The extracted data were consisted of the follow items: the first author’s name, publication year, population (Ethnicity), methods, matching criteria, total number of cases and age (years).

The quality assessment of the included trials was undertaken independently by two review authors (H.S. and L.X.Y.), following Newcastle–Ottawa Scale (NOS), which is composed of three parts: selection, comparability and exposure. It is a semi-quantitative scale, and a score of 0–9 stars was assigned to each study. A total score of <7 was considered poor and 7–9 was deemed high quality.

### Statistical analysis

We measured the statin effect on continuous outcomes (e.g. change of mean platelet volume) by SMD with 95% confidence interval (CI). We used Review manager 5.3 and Stata14.0 software to perform the meta-analysis in the present study. Sensitivity analysis was performed by changing effect model and remerging data after excluding abstracts. Begg’s test and egger’s test were used to detect the asymmetry of the funnel plot, which (*P*<0.05) were considered to be representative of statistically significant publication bias. Heterogeneity among studies was assessed by I^2^ statistic. I^2^ > 50% indicated evidence of heterogeneity. If heterogeneity existed among the studies, the random effects model was used to estimate the pooled effect size. Otherwise, the fixed effects model was adopted. Subgroup analyses regarding drug type, follow-up, sample size, ethnicity, mean age and NOS score, were also performed to explore source of heterogeneity.

## Results

### Literature search

**Figure 1:**
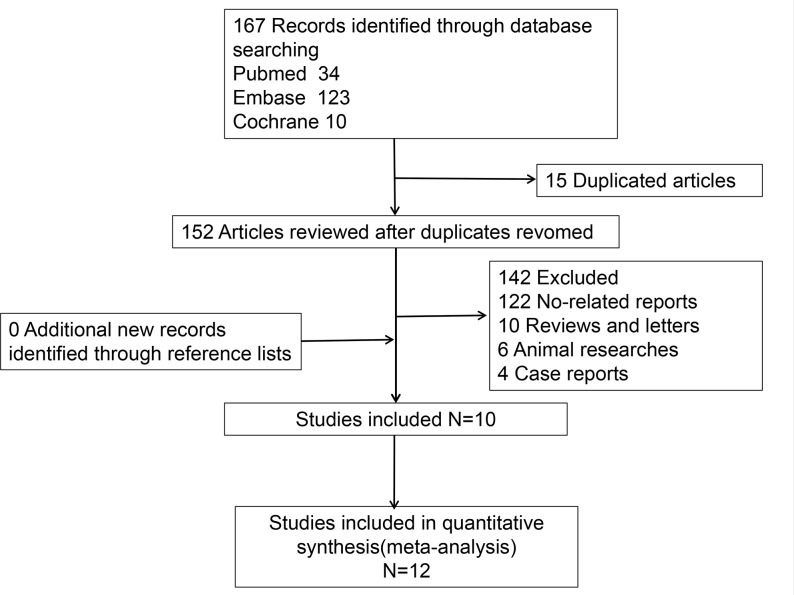
Flow diagram for literature selection

After initial retrieval, 167 articles were obtained. 15 articles were removed after repeated examination. 152 articles were excluded after reading the title and abstract, which included four case reports, six animal researches, ten reviews and letters, and 122 unrelated studies such as relationship between statin and cancers, researches about platelet function. The remaining ten articles included eight case control studies [8,10–12,14–17] and two abstracts [9,13]. Two of these studies [10,11] reported the effects of two statins on MPV, so we considered a total of 12 studies to be included in the data analysis. The flowchart of literature inclusion was shown in Figure 1.

### Characteristics of the selected studies

The characteristics included in the article are shown in Table 1. Among included studies [8–17], there were seven papers published in the past 5 years [9–11,14–17], eight papers about Caucasian [8,10,12–17], two papers about Asian [9,11], five papers with high-quality [8,10,14,15,17], two abstracts without NOS score [9,13]. Statins included atorvastatin (initial dose level to maximum dose level, 10–80 mg/d), rosuvastatin (initial dose level to excess maximum dose level, 10–40 mg/d), pravastatin (initial dose level to maximum dose level, 20–40 mg/d) and simvastatin (initial dose level, 5–20 mg/d).The number of patients ranged from 10 to 261, and there were four literatures with the number of patients greater than or equal to 100. The follow-up time span was 4–24 weeks, and the follow-up time of six articles was more than or equal to 12 weeks [8,9,13,15–17]. Risks of cardiovascular diseases mainly include: diabetes mellitus and dyslipidemia. In terms of data types, IQR of mean platelet volume was provided in two studies [16,17].

**Table 1:** Main characteristics of eligible studies

Study and year	Country	NOS	Drug	Follow-up (Weeks)	Disease	Patients	Dosage
Akin F 2013	Turkey	7	Atorvastatin	24	Hypercholesterolemia	79	10–80mg/d
Akyuz A 2015	Turkey	7	Rosuvastatin	4–12	Diabetes mellitus	178	40 mg/d
Broijersen A 1994	Sweden	6	Pravastatin	4	Hypercholesterolaemia	10	20–40 mg/d
Coban E 2007	Turkey	7	Rosuvastatin	12	Dyslipidemia	30	10 mg/d
Gungoren F 2015	Turkey	7	Rosuvastatin	24	Hypercholesterolemia	261	10–40 mg/d
			Atorvastatin				10–80 mg/d
Kucera M 2015	Slovak	6	Atorvastatin	12	Hypercholesterolaemia	40	40 mg/d
Sivri N 2013	Turkey	7	Atorvastatin	4–8	Hypercholesterolemia, diabetes mellitus	74	10–80 mg/d
			Rosuvastatin	4–8		71	10–40 mg/d
Xian-Yu JB 2015	China	6	Simvastatin	4	Hypercholesterolemia, diabetes mellitus	76	5–20 mg/d
			Atorvastatin	4		74	10–80 mg/d
Butt, N F 2018	Pakistan	No	Atorvastatin	12	Dyslipidemia	100	20 mg/d
Erdem, G 2009	Turkey	No	Rosuvastatin Atorvastatin	12	Hypercholesterolaemia	196	Unknown

**Abbreviation:** NOS, Newcastle–Ottawa Scale.

### Meta-analysis of statin therapy and MPV

**Figure 2:**
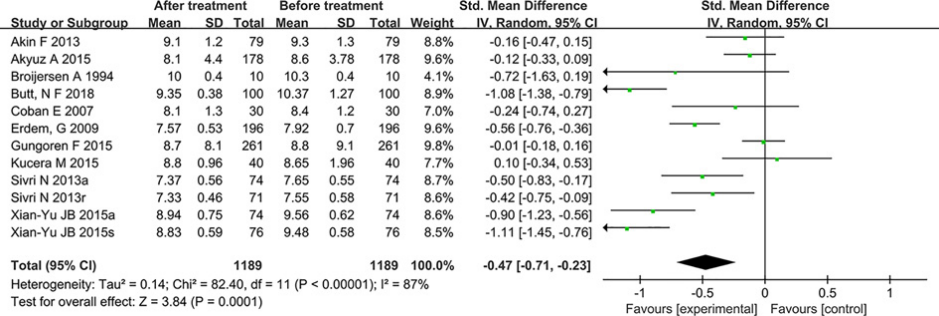
Effect of statin therapy on mean platelet volume

The pooled analysis is shown in Figure 2. The results showed that the mean platelet volume significantly down-regulated after statin treatment [SMD = −0.47 (−0.71–0.23)], which was statistically significant (*P*=0.0001). There was significant heterogeneity among studies (*P*<0.00001, I^2^ = 87%), so the random-effect model was used.

### Subgroup analysis

Subgroup analysis based on drug type, follow-up, sample size, ethnicity, mean age and NOS score, was performed and the results are shown in Table 2. In terms of specific drug administration, the mean platelet volume could be lowered by the atorvastatin [SMD = −0.52 (−0.94, −0.10)] and rosuvastatin [SMD = −0.22 (−0.40, −0.03)], and the results were statistically significant. The heterogeneity of the rosuvastatin group significantly increased (I^2^ = 9%). Likewise, results of ethnicity [Asian, SMD = −1.04 (−1.22, −0.85); Caucasian, SMD = −0.26 (−0.44, −0.08)] and NOS score group [≥7, SMD = −0.21(−0.37, −0.05); <7, SMD = −0.67 (−1.23, −0.11)] were similar to drug type, mean platelet volume showing a statistically significant down-regulation, and the heterogeneity of Asian group (I^2^ = 0%) and ≥7 group (I^2^ = 48.5%) also significantly increased. Although analysis results of follow-up [≥12 weeks, SMD = −0.34 (−0.69, −0.02); <12 weeks, SMD = −0.61(−0.95, −0.26)] and sample size group [≥100, SMD = −0.43 (−0.85, −0.01); <100, SMD = −0.49 (−0.78, −0.20)] exhibited obvious difference, heterogeneity failed to decrease significantly, which suggested both of follow-up and sample size were not the source of heterogeneity. The age range was divided by 55 years (≥55 years and <55 years), and heterogeneity of the two group showed a significant down-regulation (*I*^2^ = 0%, 64.5%). In <55 years group, statin therapy could decrease mean platelet volume [SMD = −0.66 (−0.94, −0.38)]༌which was statistically significant, but this similar result did not appear in ≥55 years group [SMD = −0.06 (−0.18, 0.06)].

**Table 2:** Subgroup analysis about effect of statin therapy on mean platelet volume

Subgroup		Standard mean difference (95% CI)	Heterogeneity	
			I^2^ (%)	*P*-value
Drug type	Atorvastatin	−0.52 (−0.94, −0.10)	87	<0.00001
	Rosuvastatin	−0.22 (−0.40, −0.03)	9	= 0.33
Follow-up	≥12 week	−0.34 (−0.69, −0.02)	90	<0.00001
	<12 week	−0.61 (−0.95, −0.26)	84	<0.00001
Sample size	≥100	−0.43 (−0.85, −0.01)	94	<0.00001
	<100	−0.49 (−0.78, −0.20)	77	<0.0001
Ethnicity	Asian	−1.04 (−1.22, −0.85)	0	= 0.663
	Caucasian	−0.26 (−0.44, −0.08)	68.4	= 0.001
Mean age	≥55 years	−0.06 (−0.18, 0.06)	0	0.675
	<55 years	−0.66 (−0.94, −0.38)	64.5	= 0.015
NOS score	≥7	−0.21 (−0.37, −0.05)	48.5	0.084
	<7	−0.67 (−1.23, −0.11)	84.5	0.000

### Sensitivity analysis

Sensitivity analysis was achieved by transforming effect model and re-synthesizing data with excluding abstracts. The combined result was statistically significance and with no significant change [SMD = −0.39 (−0.47, −0.31), *P*<0.05] after the random model was converted to a fixed model. The result of re-synthesizing data with excluding abstracts was similar [SMD = −0.39 (−0.64, −0.14), *P*<0.05]. Sensitivity analysis showed that our meta-analysis results were stable and reliable.

### Assessment of publication bias and linear correlation of MPV changes by intensity statin

**Figure 3:**
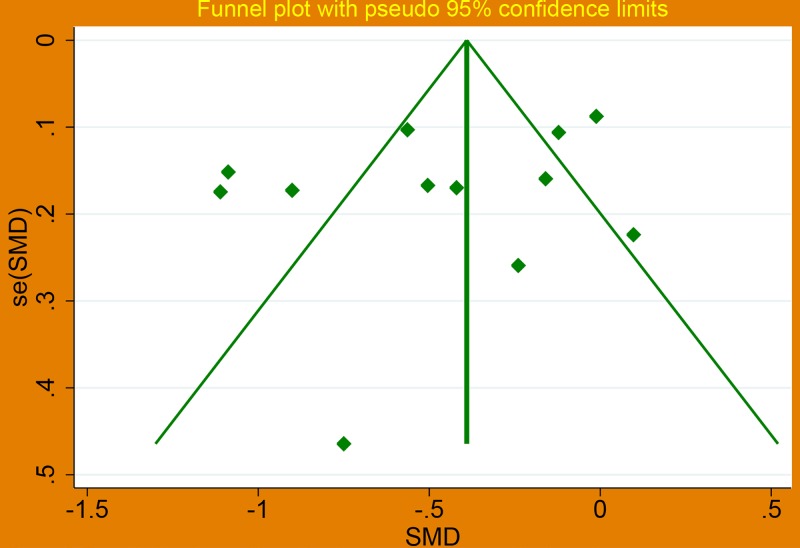
Funnel plot about effect of statin therapy on mean platelet volume

**Figure 4:**
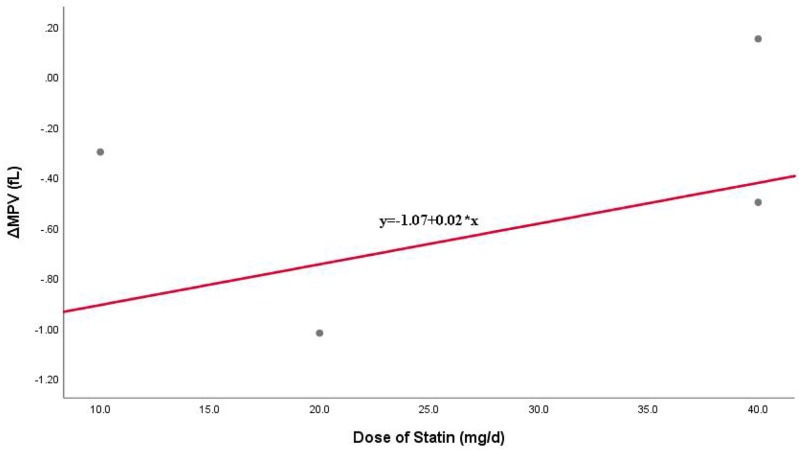
Linear correlation of MPV changes by intensity statin

A funnel plot (Figure 3), Begg’s test and Egger’s test were performed to investigate the potential publication bias. The results showed no significant publication bias existed (Begg’s *P*=0.451>0.05, Egger’s *P*=0.271>0.05). Linear correlation of MPV changes by intensity statin is shown in Figure 4, and the result suggested that there was a positive correlation.

## Discussion

Our study shows that statin do reduce mean platelet volume in patients with cardiovascular disease risk factors, which indirectly suggests that statin could inhibit platelet function. To our knowledge, this is the first study to demonstrate the effect of statin on platelets through systematic review and meta-analysis, providing a powerful supplement for the diversity of clinical effects of statin.

In clinical practice, cardiovascular disease is a kind of common and multiple diseases, with a long course and rapid progress. Platelets play an important role in the development of cardiovascular diseases, especially atherosclerosis. Cardiovascular disease is mainly caused by thrombosis. In the vascular lumen, platelet aggregation and adhesion lead to thrombosis, which causes narrowing and obstruction of the lumen, myocardial ischemia, hypoxia and eventually necrosis. Obesity, diabetes༌hypertension and dyslipidemia are risk factors for cardiovascular diseases. Many studies have shown that platelets are highly reactive in these risk factors, which in turn can accelerate the development of cardiovascular diseases [19–22]. Platelet activation is often accompanied by an increase in volume, i.e., increased MPV. Coban et al. [23] observed a mean MPV significantly higher in the group of obese women, in comparison with the non-obese group (8.18 ± 1.09 vs 8.01 ± 0.95 fL, *P*=0.004). In addition, Coban et al. [24] in another case–control study with 200 subjects and Ozkan et al. [25] in a case–control study with 108 children aged 6–16 years reached similar conclusions.

Patients with Type 2 DM have a higher risk of coagulation abnormalities and thromboembolic events [26,27], and elevated MPV and its significance as marker in Type 2 DM have been elaborated in many studies [28–31]. The same is true for those with Arterial hypertension or dyslipidemia [32,33]. Therefore, MPV is an important marker for patients with cardiovascular disease risk factors and represents abnormal platelet function. Improving platelet function and reducing MPV are important in preventing cardiovascular events

Statin can significantly reduce blood cholesterol levels and the risk of cardiovascular disease and ischemic stroke [34,35]. In addition to inhibiting lipid synthesis, statin also has antioxidant [36], anti-inflammatory [37] and antithrombotic effects [38]. Our study showed that statin can reduce MPV and regulate platelet function, which may be mainly through multiple mechanisms to combat platelet activation. CD36 (glycoprotein IV) is expressed in a variety of cells on the surface of the single glycoprotein, and ox-LDL binds to platelet membrane CD36 to generate endogenous ROS, which can promote platelet activation and thrombosis [39]. Simvastatin and pravastatin can inhibit platelet activation by directly interacting with CD36 or by affecting CD36 intracellular signal transduction pathways [38]. Peroxisome proliferator-activated receptor (PPAR) is a member of the nuclear steroid hormone receptor superfamily, and its biological functions of transcriptional regulation are mainly performed by binding specific DNA reaction elements and retinol X heterodimer receptors [40]. Simvastatin is dose-dependent on the induction of PPARα and PPARγ activation, and the up-regulation of PPARγ expression also inhibited collagen-induced plaque aggregation. Meanwhile, elevated expression of CD62, bispecific phosphatase 2 and Ca2 + mobilization induced by simvastatin could further increase antiplatelet activity [41]. Lee et al. [42] reported effect of cyclicAMP-eNOS/NO-cyclicAMP pathway in antiplatelet activity induced by statin. Activation of cyclicAMP-eNOS/NO-cyclicAMP pathway could cause phospholipase Cγ2-Protein kinase C-mitogen activated protein kinase-TXA2 cascade reactions inhibition, which suggests antiplatelet activity. Ni et al. [43] reported atorvastatin can increase the level of guanosine cyclophosphate in platelets and delay the maximum activation rate of p-selectin and CD41, which inhibited activation of platelet induced by H-Gly-Tyr-Pro-GlyLys-Phe-OH (GYP) and thrombin. Serebruany et al. [44] demonstrated that multiple statin can reduce the expression of thrombin receptor protein kinase-activated receptor-1, and reduce platelet activation and thrombosis in the evaluation of the primary prevention effect of statin therapy in patients with metabolic syndrome. Therefore, it is not hard to understand why statin could reduce MPV.

In the ten studies, we included the results of Gungoren et al. [17] and Kucera et al. [16] indicated that statin therapy may not affect MPV. After comprehensive analysis, we found that the biggest difference between these two articles and other articles is the original data type of MPV, both of two using median and IQR. For this reason, we removed these two articles and recombined the data, and the result [SMD = −0.58 (−0.82, −0.34), *P*=0.000] verified our idea to a certain degree.

Our study indicates that statin therapy could reduce MPV regardless of drug type, follow-up time, sample size, ethnicity, and <55 years subgroup, and we also find that drug type, ethnicity, age, and literature quality are sources of heterogeneity. Sensitivity analysis and publication bias test show that our results are stable and reliable. The age group ≥55 years shows no effect [SMD = −0.06 (−0.18, 0.06)], and we fail to explain it.

Of course, we need to point out the limitations of our research. First of all, the time interval of blood sample collection and analysis, and the use of different anticoagulants (such as citrate and EDTA) may be the influencing factors, but due to insufficient data, it cannot be verified by subgroup analysis; Second, the follow-up time of the included studies was relatively long, which could not guarantee that the patients did not have behaviors that interfered with the study results; Then, we could not perform subgroup analysis about pravastatin and simvastatin; Finally, there is no way to know if there is a drug dose effect because the doses overlap and cannot be analyzed further.

Fortunately, our study demonstrated that statin therapy may reduce MPV, indirectly demonstrating platelet inhibition. Given the importance of MPV as a marker of activated platelets and as a predictor of vascular events, this effect of statin therapy is encouraging. What is more, our study suggests when we use MPV to diagnose and monitor patients with cardiovascular disease, we need to pay attention to patients’ statin use.

## Abbreviations

CI: confidence interval
DM: diabetes mellitus
IQR: interquartile range
MPV: mean platelet volume
NOS: Newcastle–Ottawa Scale
ox-LDL: oxidation-low density lipoprotein
PPAR: peroxisome proliferator-activated receptor
ROS: reactive oxygen species
SD: standard difference
SMD: standard mean difference

## References

[1] Gong J. and Gu N. (2017) Analysis on correlation between blood stasis syndrome of coronary heart disease and coagulation function and blood platelet parameters. Biomed. Res. 28, 9825–9829

[2] Hudzik B., Korzonek-Szlacheta I., SzkodziåSki J., Liszka R., Lekston A., Zubelewicz-Szkodzińska B. (2018) Association between multimorbidity and mean platelet volume in diabetic patients with acute myocardial infarction. Acta Diabetol. 55, 175–183 10.1007/s00592-017-1079-6 29189913 PMC5816096

[3] Cho S.Y., You E., Lee H.J., Lee W.I. and Park T.S. (2014) Smoking cession decreases mean platelet volume in healthy Korean populations. Clin. Lab. 60, 1413–1416 10.7754/Clin.Lab.2013.130901 25185431

[4] Li G., Zhang Y.Y., Zhu Z.W. and Du J. (2017) Association between mean platelet volume and hypertension incidence. Hypertens. Res. 40, 779–784 28275234 10.1038/hr.2017.30

[5] Varol E., Aksoy F., Bas H.A., Ari H. and Ozaydin M. (2014) Mean platelet volume is elevated in patients with low high-density lipoprotein cholesterol. Angiology 65, 733–736 10.1177/0003319713504024 24065627

[6] Erdim I. and Oghan F. (2017) Blood count values and ratios for predicting sleep apnea in obese children. Int. J. Pediatr. Otorhinolaryngol. 98, 85 10.1016/j.ijporl.2017.04.043 28583511

[7] Akyuz A., Akkoyun D.C., Degirmenci H. and Alp R. (2015) Atrial fibrillation is associated with increased mean platelet volume and apnea hypopnea index in patients with obstructive sleep apnea. Angiology 66, 525–530 10.1177/0003319714548567 25163774

[8] Coban E. and Afacan B. (2008) The effect of rosuvastatin treatment on the mean platelet volume in patients with uncontrolled primary dyslipidemia with hypolipidemic diet treatment. Platelets 19, 111–114 10.1080/09537100701230444 17852772

[9] Butt N.F., Rathore R., Latif H., Mehmood H., Faisal H.S. and Iqbal A. (2018) Effect of atorvastatin on hematological parameters in patients with dyslipidemias. Pak. J. Med. Health Sci. 12, 1087–1090

[10] Sivri N., Tekin G., Yalta K., Aksoy Y., Senen K. and Yetkin E. (2013) Statins decrease mean platelet volume irrespective of cholesterol lowering effect. Kardiol. Pol. 71, 1042–1047 10.5603/KP.2013.0259 24197585

[11] Xian-Yu J.B., Feng J.F., Chen Y.C. and Yang Y.W. (2015) Effects of simvastatin and atorvastatin on biochemical and hematological markers in patients with risk of cardiovascular diseases. Int. J. Clin. Exp. Med. 8, 13983–13989 26550356 PMC4613041

[12] Broijersen A., Eriksson M., Larsson P.T., Beck O., Berglund L., Angelin B. (1994) Effects of selective LDL-apheresis and pravastatin therapy on platelet function in familial hypercholesterolaemia. Eur. J. Clin. Invest. 24, 488–498 10.1111/j.1365-2362.1994.tb02380.x 7957507

[13] Erdem G., Tasci I., Celebi G., Ozgur G., Dogru T., Sonmez A. (2009) LDL-cholesterol lowering either through therapeutic lifestyle change intervention or HMG-COA reductase inhibitor treatment decreases mean platelet volume values in people with elevated LDL-cholesterol. Atheroscler. Suppl. 10, 10.1016/S1567-5688(09)70228-4

[14] Akyüz A., Akkoyun D.Ç., Değirmenci H. and Oran M. (2016) Rosuvastatin decreases mean platelet volume in patients with diabetes mellitus. Angiology 67, 116–120 10.1177/0003319715584725 25943745

[15] Akin F., Ayça B., Köse N., Şahin I., Akin M.N., Canbek T.D. (2013) Effect of atorvastatin on hematologic parameters in patients with hypercholesterolemia. Angiology 64, 621–625 10.1177/0003319713479154 23460112

[16] Kucera M., Balaz D., Kruzliak P., Ciccocioppo R., Oravec S., Rodrigo L. (2015) The effects of atorvastatin treatment on the mean platelet volume and red cell distribution width in patients with dyslipoproteinemia and comparison with plasma atherogenicity indicators -- a pilot study. Clin. Biochem. 48, 557–561 10.1016/j.clinbiochem.2015.02.010 25727667

[17] Gungoren F., Besli F., Caliskan S., Polat U., Basar C. and Serda O.A. (2015) Statin therapy may not effect NLR and MPV levels in patients with hypercholesterolemia. Angiology 67, 536–540 10.1177/0003319715604098 26341259

[18] Higgins J. and Green S. (2008) Cochrane Handbook for Systematic Reviews of Interventions, Wiley-Blackwell, Hoboken, U.S.

[19] Santilli F., Vazzana N., Liani R., Guagnano M.T. and Davì G. (2012) Platelet activation in obesity and metabolic syndrome. Obes. Rev. 13, 27–42 10.1111/j.1467-789X.2011.00930.x 21917110

[20] Lee E.Y., Kima S.J., Song Y.J., Choi S.J. and Song J. (2013) Immature platelet fraction in diabetes mellitus and metabolic syndrome. Thromb. Res. 132, 692–695 10.1016/j.thromres.2013.09.035 24140451

[21] Ulutas K.T., Dokuyucu R., Sefl F., Yengil E., Sumbu A.T., Rizaoglu H. (2014) Evaluation of mean platelet volume in patients with type 2 diabetes mellitus and blood glucose regulation: a marker for atherosclerosis? Int. J. Clin. Exp. Med. 7, 955–961 24955167 PMC4057846

[22] Lip G.Y. (2000) Target organ damage and the prothrombotic state in hypertension. Hypertension 36, 975–977 10.1161/01.HYP.36.6.975 11116110

[23] Coban E., Ozdogan M., Yazicioglu G. and Akcit F. (2005) The mean platelet volume in patients with obesity. Int. J. Clin. Pract. 59, 981–982 10.1111/j.1742-1241.2005.00500.x 16033624

[24] Coban E., Yilmaz A. and Sari R. (2007) The effect of weight loss on the mean platelet volume in obese patients. Platelets 18, 212–216 10.1080/09537100600975362 17497433

[25] Özkan E.A., Khosroshahi H.E., Serin H.I., Özdemir Z.T., Kılıç M., Ekim M. (2015) The evaluation of carotid intima-media thickness and mean platelet volume values and correlation with cardiac functions in obese children. Int. J. Clin. Exp. Med. 8, 22557–22563 26885242 PMC4730028

[26] Kim J.H., Bae H.Y. and Kim S.Y. (2014) Response: clinical marker of platelet hyperreactivity in diabetes mellitus (diabetes metab j 2013;37:423-8). Diabetes Metab. J. 38, 160–161 10.4093/dmj.2014.38.2.160 24851211 PMC4021304

[27] Suslova T.E., Sitozhevskii A.V., Ogurkova O.N., Kravchenko E.S., Kologrivova I.V., Anfnogenova Y. (2014) Platelet hemostasis in patients with metabolic syndrome and type 2 diabetes mellitus: cGMP-and NO-dependent mechanisms in the insulin-mediated platelet aggregation. Front. Physiol. 5, 501 25601838 10.3389/fphys.2014.00501 PMC4283519

[28] Han J.Y., Choi D.H., Choi S.W., Kim B.B., Ki Y.J., Chung J.W. (2013) Stroke or coronary artery disease prediction from mean platelet volume in patients with type 2 diabetes mellitus. Platelets 24, 401–406 10.3109/09537104.2012.710858 22871068

[29] Ulutas K.T., Dokuyucu R., Sefl F., Yengil E., Sumbu A.T., Rizaoglu H. (2014) Evaluation of mean platelet volume in patients with type 2 diabetes mellitus and blood glucose regulation: a marker for atherosclerosis? Int. J. Clin. Exp. Med. 7, 955–961 24955167 PMC4057846

[30] Shimodaira M., Niwa T., Nakajima K., Kobayashi M., Hanyu N. and Nakayama T. (2013) Correlation between mean platelet volume and fasting plasma glucose levels in prediabetic and normoglycemic individuals. Cardiovasc. Diabetol. 12, 14 10.1186/1475-2840-12-14 23311535 PMC3558413

[31] Zhou Z., Chen H., Sun M. and Ju H. (2018) Mean platelet volume and gestational diabetes mellitus: a systematic review and meta-analysis. J. Diabetes Res. 2018, 1985026 10.1155/2018/1985026 29854818 PMC5954880

[32] Coban E., Yazicioglu G., Berkant Avci A. and Akcit F. (2005) The mean platelet volume in patients with essential and white coat hypertension. Platelets 16, 435–438 10.1080/09537100500163572 16236605

[33] Boos C.J., Beevers G.D. and Lip G.Y. (2007) Assessment of platelet activation indices using the ADVIATM 120 amongst ‘high-risk’ patients with hypertension. Ann. Med. 39, 72–78 10.1080/07853890601040063 17364453

[34] Amarenco P. and Labreuche J. (2009) Lipid management in the prevention of stroke: review and updated meta-analysis of statins for stroke prevention. Lancet Neurol. 8, 453–463 10.1016/S1474-4422(09)70058-4 19375663

[35] Mihaylova B., Emberson J., Blackwell L., Keech A., Simes J.and Cholesterol Treatment Trialists’ (CTT) Collaborators (2012) The effects of lowering LDL cholesterol with statin therapy in people at low risk of vascular disease: meta-analysis of individual data from 27 randomised trials. Lancet 380, 581–590 10.1016/S0140-6736(12)60367-5 22607822 PMC3437972

[36] Margaritis M. 1, Channon K.M. and Antoniades C. (2014) Statins as regulators of redox state in the vascular endothelium: beyond lipid lowering. Antioxid. Redox Signal. 20, 1198–1215 10.1089/ars.2013.5430 24111702 PMC3934595

[37] Kawabori M. and Yenari M.A. (2015) Inflammatory responses in brain ischemia. Curr. Med. Chem. 22, 1258–1277 10.2174/0929867322666150209154036 25666795 PMC5568039

[38] Luzak B., Rywaniak J., Stanczyk L. and Watala C. (2012) Pravastatin and simvastatin improves acetylsalicylic acid-mediated *in vitro* blood platelet inhibition. Eur. J. Clin. Invest. 42, 864–872 10.1111/j.1365-2362.2012.02661.x 22409214

[39] Magwenzi S., Woodward C., Wraith K.S., Aburima A., Raslan Z., Jones H. (2015) Oxidized LDL activates blood platelets through CD36/NOX2-mediated inhibition of the cGMP/protein kinase G signaling cascade. Blood 125, 2693–2703 10.1182/blood-2014-05-574491 25710879 PMC4408294

[40] Yano M., Matsumura T., Senokuchi T., Ishii N., Murata Y., Taketa K. (2007) Statins activate peroxisome proliferator-activated receptor gamma through extracellular signal-regulated kinase 1 /2 and p38 mitogen-activated protein kinase-dependent cyclooxygenase-2 expression in macrophages. Circ. Res. 100, 1442–1451 10.1161/01.RES.0000268411.49545.9c 17463321

[41] Du H., Hu H., Zheng H., Hao J., Yang J.C. and Cui W. (2014) Effects of peroxisome proliferatoractivated receptor gamma in simvastatin antiplatelet activity: Influences on cAMP and mitogen-activated protein kinases. Thromb. Res. 134, 111–120 10.1016/j.thromres.2014.05.005 24856644

[42] Lee Y.M., Chen W.F., Chou D.S., Jayakumar T., Hou S.Y., Lee J.J. (2010) Cyclic nucleotides and mitogen-activated protein kinases: regulation of simvastatin in platelet activation. J. Biomed. Sci. 17, 45 10.1186/1423-0127-17-45 20525309 PMC2894762

[43] Ni R., Peleg T. and Gross P.L. (2012) Atorvastatin delays murine platelet activation *in vivo* even in the absence of endothelial NO synthase. Arterioscler. Thromb. Vasc. Biol. 32, 2609–2615 10.1161/ATVBAHA.112.300090 22995523

[44] Serebruany V.L., Miller M., Pokov A.N., Malinin A.I., Lowry D.R., Tanguay J.F. (2006) Effect of statins on platelet PAR-1 thrombin receptor in patients with the metabolic syndrome (from the PAR-1 inhibition by statins [PARIS] study). Am. J. Cardiol. 97, 1332–1336 10.1016/j.amjcard.2005.11.058 16635606

